# Case of Inflamed Testis, in Which an Extraordinary Quantity of Blood Was Taken, and Purgatives Exhibited Freely

**Published:** 1816-09

**Authors:** W. S. Clark

**Affiliations:** Member of the Royal College of Surgeons, and Late Resident Surgeon of the Lock Hospital, London.


*For the London Medical and Physical Journal.*

### *Case of inflamed Testis, in which an extraordinary quantity of Blood was taken, and Purgatives exhibited freely;* by W. S. Clark, Esq. Member of the Royal College of Surgeons, and late resident Surgeon of the Lock Hospital, London.

THE following case is not presented to the public merely as a novelty, but to shew to what an extent general depletion is sometimes necessary to be carried, even in parts exterior to the body, and not essentially necessary to the functions of life, in order to save those parts from threatened destruction. And, here, I cannot proceed without just observing how much each member of the medical profession must have cause to congratulate himself upon the great and valuable valuable improvements that have lately been made in this branch of medical science, by men of our own day, and of our own country, where the skill and abilities of its professors have long reached the highest pinnacle of fame. How many diseases of this country, that for ages have bid defiance to every attempt to subdue them, have now found an almost certain antidote, if early and judiciously applied, in a powerful system of depletion; and none more conspicuous than that formidable and fatal disease to which lying-in women are at certain periods liable to, viz. the puerperal fever connected with peritoneal inflammation—a disease which has caused more contrariety of opinion, and more diversity of practice, with as little corresponding success, as any which the history of medicine affords. This disease, however, as far as our present limited experience will allow us to judge, appears to be now successfully combated, and the valuable work upon this subject lately sent forth from the pen of Dr. Armstrong, will convey to posterity a system of practice highly calculated to form a basis for further investigation by every succeeding practitioner; but, if in this favoured country diseases have existed which have hitherto baffled human skill, and required all the improvement that medical science could suggest, how much more has this been necessary on the inhospitable shores of foreign climes, where numbers of our brave countrymen have so long exposed their lives, and been subjected to the attacks of diseases mortal almost at their commencement, resisting every attempt that could be devised to stem their impetuous progress, and quickly hurling the unfortunate sufferer out of existence. These, however, now become, literally speaking, inoffensive, and sink into comparative insignificancy, at the approach, and upon the application, of that potent and triumphant instrument—the lancet.

These observations are offered as introductory to the following history of inflammation of the testis, a case common in its occurrence, but remarkably severe in this instance, both in its resistance to the means made use of, and the unusual size the part had acquired before the reduction of the acute stage was effected. The subject was a robust and plethoric person in this city, who applied to me, and who stated, that some little time before he had contracted a gonorrhœa, for which he was recommended the use of a strong astringent injection; by this means the *gonorrhœal discharge was effectually restrained*, but was followed by a metastasis of the diseased action to the testis, with all the usual symptoms of acute pain, swelling, thickening of the spermatic chord, pain in the groin and

 back, back, and fever; having endured all this suffering for two or three days, he applied for relief. Finding the symptoms increasing rapidly and threatening serious mischief, I instantly directed for him the following plan:

June 24, 1816.—V. S. ad ℥xxx. vel ad syncopen.

℞. Lotio. Ceruss. Acet. part. affect. ap.

℞. Hydr. Submuriatis, gr. viii.

Ft. Bol. h. s. s.

℞. Haust. Cathart. ā Sulph. Mag. mane Sumend.

These evacuating means were followed up the next day by a simple nitrous powder.

℞. Nitri. Purif. ℈j.

Pulv. Tragacanth. c. ℈ſs.

M. ft. Pulv. 4tis horis sum. in Decoct. Hordei.

A strict antiphlogistic regimen was likewise enjoined.

June 26th.—The symptoms have not at all subsided; the pain is excessive, testicle increasing in size, and constitutional irritation severe.

V. S. rep. ad ℥xxx.

Bol. Hydr. Submur. rep. et Haust. Cathart.

Cont^r. Pulv. Nitros. et Lotio. Ceruss. Acet.

30th.—From the 26th to this time, the symptoms have continued nearly stationary, but on this day have become considerably aggravated. The testicle is enormously swelled, the inflammation extending up the chord, great tenderness of the abdominal parietes round the ring, and considerable pain in the back.

V. S. statim ad ℥xxxv. vel ad syncopen.

Rep^r. Bol. Hyd. Submur. adde

Opii. Pulv. gr. iſs. M. h. s. s.

℞. Mist. Cathart. ℥vj.

Mag. Sulph. ʒx.

Tinct. Jalapæ, ʒiſs. M. sum. Cochl. iij. om. hor.

Cont^r. Lotio. Repig. et Pulv. Nitros.

And an uninterrupted recumbent posture.

July 1st.—After the last powerful bleeding, considerable relief seemed to be obtained; the refrigerent and the cathartic medicines have alternately been exhibited, and a strict antiphlogistic regimen attended to.

July 3d.—This morning a considerable increase of the pain and inflammation has come on, and the size of the testis so large as to give some reason to fear its terminating in an entire suppuration.

V. S. rep. ad ℥xvj.

Applic^r. Hirud. No. x. part. Dolent.

Cont. Bol. Cathart. et Mist.

5th.—

5th.—Symptoms not worse, nor much increase in the size of the testis.

Hirud. No. x. repr. contr. Medicamenta.

7th.—I was agreeably surprised this morning to find a gradual but decided improvement in all the symptoms, the pain and heat in the testis greatly relieved, the part somewhat softer, and the patient in a composed state.

10th.—All has gone on favourably since the last report.

20th.—From this time every unfavourable symptom has gradually subsided, the testicle, with its spermatic chord, slowly approaching to its natural state, and the patient, although he lost in a short time upwards of 110 oz. of blood, [besides what was taken by the leeches,] sufficiently able to be removed into the country for the final recovery of his health.

*York; July* 23, 1816.

